# Is the aging human ovary still ticking?: Expression of clock-genes in luteinized granulosa cells of young and older women

**DOI:** 10.1186/s13048-018-0471-3

**Published:** 2018-11-21

**Authors:** Amnon Brzezinski, A. Saada, H. Miller, NA Brzezinski-Sinai, A. Ben-Meir

**Affiliations:** 1 Department of Obstetrics and Gynecology, The Hebrew University Hadassah Medical Center, Jerusalem, Israel; 2 Department of Genetics & Metabolism, The Hebrew University Hadassah Medical Center, Jerusalem, Israel

**Keywords:** Circadian clock genes, Granulosa cells, Reproductive aging

## Abstract

**Background:**

It has been shown – mostly in animal models - that circadian clock genes are expressed in granulosa cells and in corpora luteum and might be essential for the ovulatory process and steroidogenesis.

**Objective:**

We sought to investigate which circadian clock genes exist in human granulosa cells and whether their expression and activity decrease during aging of the ovary.

**Study design:**

Human luteinized granulosa cells were isolated from young (age 18–33) and older (age 39–45) patients who underwent in-vitro fertilization treatment. Levels of clock genes expression were measured in these cells 36 h after human chorionic gonadotropin stimulation.

**Methods:**

Human luteinized granulosa cells were isolated from follicular fluid during oocyte retrieval. The mRNA expression levels of the circadian genes *CRY1, CRY2, PER1, PER2, CLOCK, ARNTL, ARNTL2*, and *NPAS2* were analyzed by quantitative polymerase chain reaction.

**Results:**

We found that the circadian genes *CRY1, CRY2, PER1, PER2, CLOCK, ARNTL, ARNTL2*, and *NPAS2*, are expressed in cultured human luteinized granulosa cells. Among these genes, there was a general trend of decreased expression in cells from older women but it reached statistical significance only for *PER1* and *CLOCK* genes (fold change of 0.27 ± 0.14; *p* = 0.03 and 0.29 ± 0.16; *p* = 0.05, respectively).

**Conclusions:**

This preliminary report indicates that molecular circadian clock genes exist in human luteinized granulosa cells. There is a decreased expression of some of these genes in older women. This decline may partially explain the decreased fertility and steroidogenesis of reproductive aging.

## Introduction

Many physiological processes and behaviors in mammals are rhythmic. These rhythms are controlled by an endogenous molecular clock within the suprachiasmatic nucleus (SCN), located in the forebrain of mammals, which is entrained by the light/dark cycle [1–4]. The SCN synchronize countless subsidiary oscillators existing in the peripheral tissues throughout the body [5, 6]. The basis for maintaining the circadian rhythm is a molecular clock consisting of interlocked transcriptional/ translational feedback loops. The proteins encoded by the genes circadian locomotor output cycles kaput (*Clock*) and brain and muscle arnt-like protein 1(*Bmal1*, also known as *ARNTL1* or *Mop3*) heterodimerize and promote the rhythmic transcription of the period (*Per1, Per2*) and cryptochrome (*Cry1, Cry2*) gene families, whereas modified PER–CRY complexes repress the activity of the CLOCK–BMAL1 complex. Over several hours, PER–CRY complexes are degraded, and the CLOCK–BMAL1 complex is eventually released from feedback inhibition [7].

There is increasing interest in the role of circadian rhythmicity in the control of reproductive function in animals and humans [2, 8]. In mammals, circulating gonadotropin luteinizing hormone (LH) and follicle-stimulating hormone (FSH) levels oscillate with a diurnal rhythm marked by afternoon surges on the day of ovulation [9, 10].

Circadian rhythms and clock genes appear to be involved in optimal reproductive performance, [11]. Expression of circadian genes *Per2* and *Bmal1* was observed in corpora luteum in rat ovaries by in situ hybridization [12]. In addition, circadian clock genes *Per2* and *Clock* were found to be involved in the regulation of steroid production and cell proliferation in granulosa cells, which turn into granulosa lutein cells after ovulation [9, 13].

Most of what we currently know regarding clock function in the mammalian ovary relates to the timing of gene expression in mature or luteinized GCs from rats and mice. We therefore sought to investigate which of the clock genes are expressed in human granulosa cells and whether ovarian aging is associated with decreased expression of these genes.

## Materials and methods

### Subjects

Young women (33 YO or younger) and older women (39 YO or older) were asked to participate in this study. The Hadassah-Hebrew University Medical Center Institutional Review Board approved this study. All subjects gave written informed consent to participate in the study.

### Luteinized granulosa cells isolation

All our subjects kept a regular and similar sleep-wake cycle. All women had standard short agonist or antagonist protocol. The treatment protocols were equally distributed in both groups (see Table 1). All samples were collected at the same time frame (between 8:30 and 10:00 am). After egg retrieval and oocyte isolation from all follicles, follicular fluid was centrifuge and top layer of pellet was collected. Granulosa cells were separated from RBCs and most WBCs by centrifuge with Lymphoprep™ (Alere Technologies, Oslo, Norway). Cells were washed two times with 1xPBS, lysed with 300 μl of RNA buffer (Zymo Research, Irvine CA, USA) and kept in − 80 until RNA isolation.

**Table 1:** Patients characteristics in young and old groups [mean (range)]

	Young group (*n* = 5)	Old group (*n* = 5)
Age	27.9 (24–29)	40.3 (39–43)
IVF indication	Male factor or mechanical	Age-related
Day 3 FSH	6.0 (4.9–7.2)	8.6 (5.8–11.3)
No. of oocyte retrieved	16.2 (10–21)	6.8 (4–9)
Ovarian stimulation protocol		
Antagonist	3/5	2/5
Short agonist	2/5	3/5

### Gene expression analysis

Gene expression analysis was performed by quantitative reverse transcription-PCR (RT-qPCR). Total RNA was isolated using Quick RNA MicroPrep (Zymo Research, Irvine CA,USA) and c-DNA was generated using qScript cDNA Synthesis kit (Quanta Biosciences, Gathersburg, MD, US). Real-time PCR (RT-PCR) was performed using Taqman Gene Expression Assays (*CRY1* Assay ID; Hs00172734_m1, *CRY2* Assay ID; Hs00323654_m1, *CLOCK* Assay ID; Hs00231857_m1, *PER1* Assay ID; Hs0001092603_m1, *PER2* Assay ID; Hs00256143_m1, *ARNTL* Hs00154147_m1, *ARNTL2* Hs00368068_m1 *NPAS2* Hs00231212_m1 from Applied Biosystems, ThermoFisher Scientific, Waltham, MA USA). Samples were run on the ABI PRISM7900HT sequence detection system (Applied Biosystems, Foster City, CA USA). Relative quantitation was calculated by the 2^-ddCT method relative to human housekeeping gene *POLR2A* (Assay ID; Hs.PT.58.25515089) (Integrated DNA Technologies, Inc. (Coralville, Iowa USA).

## Results

Five young women and five older women were enrolled in this study. Indication for in-vitro fertilization treatment included unexplained, mechanical or male infertility. As expected, young women had lower day 3 FSH and higher number of retrieved oocytes (Table 1).

**Fig. 1:**
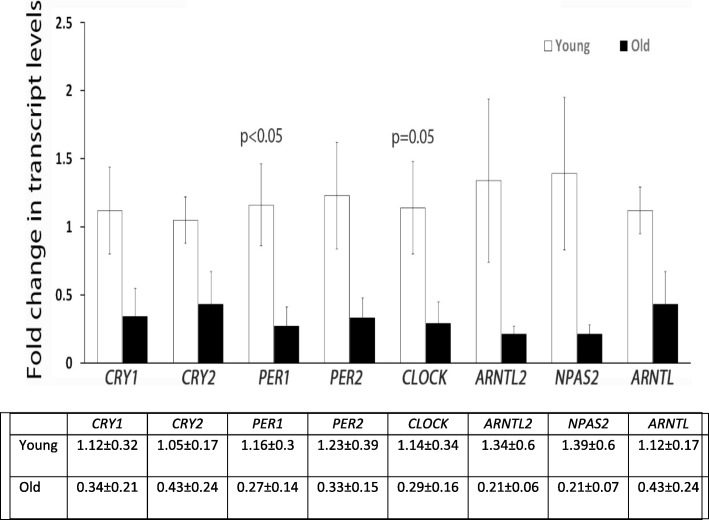
Fold change in mRNA levels of genes involved in circadian rhythm in luteinized granulosa cells were normalized to young age. Transcripts encoding *CLOCK* and *PER1* genes are reduced with aging. Each sample contained a pool of granulsa cells from several follicles (young *n* = 5, old *n* = 5)

Fold changes of mRNA levels of the CLOCK genes are presented in Fig. 1. All genes are expressed in human luteinized granulosa cells. All examined genes show tendency of decrease expression with aging, but it reached statistical significance only for *PER1* and *CLOCK* genes (fold change of 0.27 ± 0.14; *p* = 0.03 and 0.29 ± 0.16; *p* = 0.05, respectively).

## Discussion

Our results indicate that the circadian genes *CRY1, CRY2, PER1, PER2, CLOCK, ARNTL, ARNTL2*, and *NPAS2* are all expressed in cultured *human* luteinized granulosa cells. Among these genes, there was a general trend of decreased expression in cells from older women, but it reached statistical significance only for *PER1* and *CLOCK* genes.

In recent years much information has accumulated to support the importance of the clockwork mechanism in reproduction by using mutant mouse models with various disruptions of the molecular clockwork (3). The mammalian period paralogues Per1 and Per2 seem to be part of the molecular network involved in the repression of G1-S transition, while the circadian transcrip-tion factors BMAL1 and CLOCK take part to the molecular network that regulates G2-M transition. Per1 and Clock1involvement in the cell cycle control have been confirmed in diurnal low vertebrates such as the zebrafish.

There are other reports suggestive of interactions between clock genes and reproduction. For example, the report that estradiol and progesterone are involved in modification of circadian rhythm via direct regulation of the expression of clock genes [14], or the finding that LH surge apparently induces change in gene expression within the GCs of the preovulatory follicle [15]. It has also been reported recently [16] that the clock gene Bmal expression is affected by human chorionic gonadotropin (hCG) administration.

In spite of all these reports the extent to which the circadian timing system affects *human* reproductive performance is still not clear. There is only one report [17] that the circadian genes *CLOCK*, *PER2*, and *BMAL1* were found to be expressed in cultured *human* luteinized granulosa cells. They found that among these genes, only expression of *PER2* displayed oscillating patterns with a 16-h period. *CLOCK* and *BMAL1* did not show significant oscillating patterns. They also report that expression of the steroidal acute regulatory protein (STAR) gene showed an oscillating pattern that was similar to that of *PER2*.

In the present study we demonstrated the existence of all the known molecular circadian clocks in human granulosa cells. We observed a decreased expression of these genes in granulosa cells obtained from older woman as compared to young women. These preliminary findings, together with the reports about the involvement of clock genes in ovarian steroidogenesis, suggest that gradual disruption of circadian rhythm with age might lead to dysregulation of steroidogenesis in corpora luteum of the human ovary and contribute to follicular dysfunction.
